# Surface Acoustic Wave Devices: New Mechanisms, Enabling Techniques, and Application Frontiers

**DOI:** 10.3390/mi17040494

**Published:** 2026-04-17

**Authors:** Hongsheng Xu, Xiangyu Liu, Weihao Ye, Xiangyu Zeng, Akeel Qadir, Jinkai Chen

**Affiliations:** 1 Industry-Education-Research Institute of Advanced Materials and Technology for Integrated Circuits, Anhui University, Hefei 230601, China; 2 Hangzhou Institute of Technology, Xidian University, Hangzhou 311200, China; 3 School of Information Engineering, Xi’an Eurasia University, Xi’an 710065, China; akeelqadir@eurasia.edu; 4 Ministry of Education Key Laboratory of RF Circuits and Systems, Hangzhou Dianzi University, Hangzhou 310018, China

**Keywords:** surface acoustic waves (SAWs), optomechanical coupling, Brillouin scattering/optomechanics, acoustoelectric coupling, 2D materials and MXenes, non-piezoelectric substrates, lab-on-a-chip microfluidics, hybrid quantum acoustic–photonic systems

## Abstract

Surface Acoustic Wave (SAW) technology, long central to analog signal processing and RF filtering, is undergoing a major renewal. Driven by advances that decouple SAWs from traditional piezoelectric materials and fixed-function devices, the field is gaining unprecedented control over acoustic, optical, and electronic interactions at the micro and nanoscale. This review synthesizes these developments across four fronts: new physical mechanisms for SAW manipulation, emerging material platforms, ranging from thin films to 2D systems, along with reconfigurable device architectures and circuits, and the expanding landscape of applications they enable. Optical methods are reshaping how SAWs are generated and controlled, bypassing the limits of conventional electromechanical coupling. Coherent optical excitation of high-Q SAW cavities via Brillouin-like optomechanical interactions now grants access to modes in non-piezoelectric substrates such as diamond and silicon, while on-chip SAW excitation in photonic waveguides through backward stimulated Brillouin scattering opens new integrated sensing routes. In parallel, magneto-acoustic experiments have revealed nonreciprocal SAW diffraction from resonant scattering in magnetoelastic gratings. On the device side, ZnO thin-film transistors integrated on LiNbO_3_ exploit acoustoelectric coupling to realize voltage-tunable phase shifters; UHF Z-shaped delay lines achieve high sensitivity in a compact footprint; and parametric synthesis of wideband, multi-stage lattice filters targets 5G-class performance. Atomistic simulations show that SAW propagation in 2D MXene films can be engineered via surface terminations, while aerosol jet printing and SAW-assisted particle patterning provide agile, cleanroom-light fabrication of microfluidic and magnetic components. These advances enable applications ranging from hybrid quantum systems and quantum links to lab-on-a-chip particle control, SBS-based and UHF sensing, reconfigurable RF front-ends, and soft robotic actuators based on patterned magnetic composites. At the same time, optical techniques offer non-contact probes of dissipation, and MXenes and other emerging materials open new regimes of acoustic control. Conclusively, they are transforming SAW technology into a versatile, programmable platform for mediating complex interactions in next-generation electronic, photonic, and quantum systems.

## 1. Introduction

Surface acoustic waves (SAWs) have progressed from fixed-function RF filters to a broadly enabling platform spanning communications, sensing, microfluidics, advanced manufacturing, and emerging quantum interfaces. In SAWs, elastic energy is bound to a free surface or interface: Rayleigh waves travel along a single surface, Lamb waves between parallel surfaces, and Stoneley waves at solid–solid interfaces [1,2,3]. In general, acoustic wave propagation in solids can be broadly classified into surface and bulk modes depending on energy confinement. SAWs are elastic waves propagating along the surface of a substrate, with their amplitude decaying exponentially into the material such that most of the energy is confined within approximately one wavelength beneath the surface. In contrast, bulk acoustic waves (BAWs) propagate through the entire volume of the material, allowing acoustic energy to travel through the thickness of the substrate. In piezoelectric media, both SAWs and BAWs can be electrically excited via electromechanical coupling, and their propagation characteristics are governed by the elastic constants, piezoelectric coefficients, and crystallographic orientation of the substrate. Despite configuration-dependent dispersion, SAWs share elliptical particle motion and strong amplitude attenuation with depth [4], which confers long on-surface propagation and exceptional sensitivity to environmental modulation and surface functionalization traits long exploited in analog signal processing [5,6], chemical sensing [7,8], and, more recently, quantum control and single-electron transport [9,10].

While authoritative accounts cover classical SAW filters, piezoelectric transduction, and acoustofluidics, comprehensive treatments that integrate (i) symmetry-enabled transport (e.g., magnetoelastic nonreciprocity), (ii) coherent optical access to SAWs (Brillouin-like cavity coupling and on-chip SAW-SBS), and (iii) cleanroom-light/additive fabrication and printing routes alongside reconfigurable CMOS-compatible acoustoelectrics are scarce. This review fills that gap by placing these advances on a common footing mechanism → material/fabrication → device/architecture → system, highlighting design trade-offs, performance ceilings, and deployment constraints.

The angular momentum of SAWs ($L_{SAW}$) arises from the elliptical particle motion at the surface, leading to a rotational component of energy flow associated with the wave. Because SAWs superpose longitudinal and transverse polarizations, their near-surface motion carries $L_{SAW}$ that, in isotropic media, follows $L_{SAW}\propto k\;\times \;n$; this relation largely persists in anisotropic crystals such as LiNbO_3_ [11,12,13]. In media with broken time-reversal (TRS) and spatial inversion symmetries (SIS), waves exhibit directional anisotropy, and transport amplitudes differ upon reversing propagation [14]; beyond nonreciprocal propagation, SAWs can also exhibit nonreciprocal diffraction, where intensity depends on the sign of ΔQ=q−k, a phenomenon long known in optics [15,16] and recently extended to magnetoelastically patterned SAW platforms. These symmetry-enabled degrees of freedom foreshadow acoustic isolators, circulators, and robust beam steering for microwave and quantum systems.

In parallel, a new family of coherent optical interfaces is decoupling SAW operation from piezoelectric electrodes and interdigital transducers (IDTs). Electrically driven SAW devices remain essential to modern communications [17,18] and chemical/biological sensing [19,20] and SAWs have emerged as a resource for quantum systems owing to low loss, tight surface confinement, and strong coupling to disparate qubits [10,21,22,23]. Robust optical access would unlock techniques from cavity optomechanics quantum transduction [24,25], quantum-limited force/displacement sensing [25,26,27,28,29], non-classical state generation [30,31,32] and ground-state cooling [33,34,35,36] and provide an optical pathway for long-distance quantum links [37,38,39]. Non-collinear Brillouin coupling refers to an optomechanical interaction in which two optical waves incident at different angles exchange energy via a phase-matched acoustic wave defined by their wavevector and frequency difference. A frequency-tunable, Brillouin-like, non-collinear optomechanical coupling now demonstrates direct, coherent, power-tolerant access to Gaussian SAW cavities on simple single-crystal substrates, achieving record quality factors ${\approx 10}^{5}$ at cryogenic temperatures) near 500 MHz and GHz-class tunability without piezoelectricity or IDTs. Complementing this cavity route, on-chip SAW-stimulated Brillouin scattering (SAW-SBS) in highly nonlinear chalcogenide waveguides establishes coherent, all-optical excitation and readout of SAWs on integrated photonic chips. Here, SBS couples guided optical modes to surface acoustic modes, enabling narrowband filtering and surface-sensitive metrology in platforms that otherwise lack core acoustic confinement. SAW-SBS leverages the large Brillouin gain and narrow linewidth of chalcogenides to realize strong on-chip gain and MHz-scale acoustic resonances, while exploiting the inherently lower SAW frequencies for even higher frequency resolution relative to bulk acoustic interactions.

Materials-by-design is the second pillar of this transformation. Two-dimensional materials provide atomic-scale levers for dispersion, confinement, and coupling. Within 2D platforms, graphene and hBN deliver SAW-induced electromechanical responses and bandwidth extension [40,41,42,43]. A distinct class, MXenes $M_{n+1}X_{n}T_{x}$, offers tunability via surface terminations (–O, –F, –OH) and layer stacking [44,45,46]. Atomistic simulations on $T_{3}C_{2}T_{x}$ show that SAW propagation velocity is highly sensitive to both termination and thickness: oxygen-terminated monolayers support markedly higher speeds than fluorine-terminated analogues due to strengthened intralayer bonds; adding layers introduces interlayer vibrational modes, enhances dispersion, and reduces coherence, while pushing acoustic energy toward the topmost surface as thickness approaches the SAW wavelength. These sensitivities identify termination/stacking as practical design knobs for MXene-based SAW devices and suggest using SAW metrics as in situ probes of MXene structure and environment.

Acoustic streaming is the steady fluid flow generated by the dissipation of acoustic wave energy, through which propagating waves transfer momentum to the liquid and induce bulk motion and vortical motion, enabling manipulation of fluids and suspended particles. This dissipative process can also lead to acoustic heating, as part of the acoustic energy is converted into thermal energy within the fluid. Acoustic streaming can be broadly classified into boundary-driven (Rayleigh) streaming, arising from viscous effects near solid–fluid interfaces, and bulk (Eckart) streaming, which originates from attenuation of acoustic waves within the fluid. A third driver is additive, mask-lite manufacturing for acoustofluidics. SAW microfluidics has surged across biology, medicine, engineering, and materials science [47,48,49,50,51,52]. SAWs launched by applying a potential to on-substrate IDTs inject acoustic radiation and streaming forces into microdomains for precise, contactless, biocompatible manipulation. Applications span sample enrichment [53,54,55], extracellular vesicle fractionation [54,56,57,58,59,60] cell–cell interaction studies [61,62], sorting [63,64,65], stimulation [63,64,65], patterning [66,67,68,69,70], droplet manipulation and atomization [71,72,73,74], precise on-demand generation, splitting, and high-speed sorting of droplets [75,76,77] intra droplet material handling [78,79,80,81,82], intracellular delivery [83,84], and manipulation of larger organisms [85,86,87]. Conventional photolithography/evaporation/lift-off demands cleanrooms and can be incompatible with chemically sensitive substrates [88,89,90,91,92]. Aerosol jet printing overcomes these barriers with maskless, direct-write electrodes at low cost, high resolution, and without post-processing [91], printable on heated, chemically sensitive, flexible/curved substrates [93,94] with recyclable/biocompatible transparent inks [93,95,96]. Moving beyond sensors [97,98,99,100,101,102,103,104,105] recent work demonstrates the first aerosol-jet-printed IDTs on 128° Y-cut LiNbO_3_ (silver nanowires, graphene, PEDOT:PSS) operating at 5–20 MHz, shrinking per-IDT fabrication from ~40 h to ~5 min and validating acoustic streaming and particle concentration. A rapid, single-step route to SAW microfluidics.

Acoustofluidic atomization highlights how geometry and liquid supply shape performance. Atomization produces micron-scale aerosols via external energy [106]; SAW atomizers focus energy at 10–100 MHz [107,108] and, despite nanometer amplitudes, can reach boundary accelerations of $10^{7}\;m/s^{2}$ [109], enabling monodisperse aerosols without moving parts/nozzles for inhalation therapies and vaccine delivery [110,111]. Paper-strip capillary feeding is simple and power-free [112,113], but placing the strip on the chip leaks acoustic energy, distorts the meniscus, broadens size distributions (multi-peak with large-droplet jets) [114,115,116], and elevates local temperature via acoustic streaming, risking IDT damage and chip cracking [117,118,119]. A paper-strip-located-at-edge (PSLEA) method forms a short thin film at the chip edge, physically separating aerosols from jetting droplets at startup and rapidly yielding a stable, directional, uniform, convergent plume perpendicular to the surface. A handheld device based on PSLEA achieves a unimodal particle-size distribution with a median of 3.95 µm, meeting lung-inhalation requirements and advancing commercial SAW atomization.

Finally, SAWs are enabling wireless, optically compatible glazing that merges sensing and actuation. Fogging, condensation, icing, and frosting degrade visibility and safety in lenses, windscreens, aircraft windows, traffic lights, and solar panels [120,121,122], especially when surfaces at/below the dew point face high RH in cold conditions [123,124]. Many active/passive mitigations carry energy overheads, pollution/corrosion risks, or durability issues [123,125,126,127,128,129,130]; sensing is also hard because temperature, humidity, and pressure co-vary, so most methods infer phase change from shifts in mass loading, dielectric/inductive/conductive/optical properties, or vibration frequency [131]. Thin-film SAW devices on glass can simultaneously defog/de-ice and monitor: SAW streaming clears condensate [131,132,133]; interfacial vibration and localized acousto-thermal effects delay/stop frost and promote interfacial cracking/separation for de-icing; and frequency shifts track state changes in situ [132,133,134]. Because cabling to embedded optics/windows is impractical, wireless power transfer (WPT) becomes crucial [134,135,136,137,138]. Prior SAW–WPT studies enhanced piezo-tube rotation versus SAW loss [139] and demonstrated passive wireless sensing with a triboelectric nanogenerator, RF reader, and SAW resonator [140], but WPT-driven thin-film SAWs have rarely been optimized for icing protection. A resonantly tuned WPT link co-designed to the SAW resonance enables wireless defogging/de-icing with integrated frequency-shift monitoring on glass, validating performance and quantifying temperature/electrical/mass-loading contributions under cold-chamber conditions.

We synthesize advances across four axes: (i) mechanisms (magnetoelastic nonreciprocity; Brillouin-like cavity coupling and SAW-SBS for optical access; acoustoelectric control), (ii) materials and fabrication (thin-film LiNbO_3_, Sc-doped AlN, 2D materials including MXenes, and additive/cleanroom-light processes), (iii) devices and architectures (lattice-type wideband filters for 5G/6G, ultra-short-pitch and novel IDTs, electrically reconfigurable phase shifters and metasurfaces), and (iv) applications and systems (wirelessly powered, optically compatible defog/de-ice glazing; precision atomizers and microfluidics; distributed sensing; quantum-ready links). We include works that establish a new physical mechanism, materially new fabrication route, or system-level advance with quantified metrics; we exclude BAW-only topics, purely bulk ultrasonics, and RF IC design unrelated to acoustic transduction. Section 1 treats mechanisms; Section 2 materials/fabrication; Section 3 devices/architectures; Section 4 systems; Section 5 outlines cross-cutting challenges, benchmarking needs, and a roadmap to interoperable acoustic chips that complement electronics and photonics.

**Figure 1:**
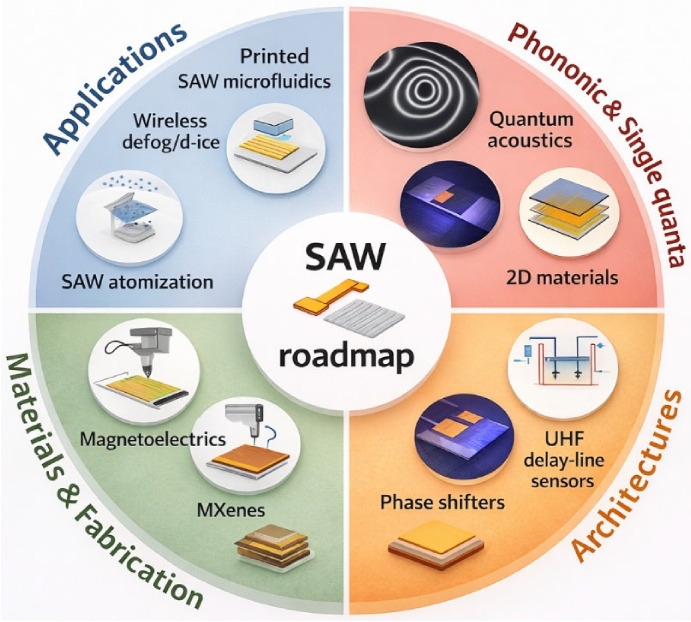
A conceptual roadmap of modern SAW technology spanning four major domains, Applications, Materials and Fabrication, Devices and Architectures, and Quantum and Single-Quantum Systems, illustrating the progression from functional devices to emerging quantum and material-driven directions.

Figure 1 provides an integrated roadmap of the rapidly expanding landscape of SAW research, illustrating how advances in applications, materials, device architectures, and quantum-scale phenomena are converging to redefine the capabilities of SAW platforms. The Applications quadrant highlights system-level implementations such as wireless defogging and de-icing, printed microfluidics, and SAW atomization that demonstrate SAWs as versatile actuators and sensors. The Materials and Fabrication quadrant captures emerging material platforms including magnetoelectrics, MXenes, and additive-printed electronics that enable flexible, tunable, and low-cost SAW devices. The Devices and Architectures quadrant showcases next-generation components such as reconfigurable phase shifters and ultra-high-frequency delay-line sensors, which extend SAW functionality into adaptive and miniaturized circuits. Finally, the Quantum and Single-Quantum Systems quadrant emphasizes the role of SAWs in quantum acoustics, phononic coupling, and 2D quantum materials, signaling their growing relevance in hybrid quantum technologies. Together, these domains position SAWs as a unifying platform bridging classical signal processing, emerging materials science, and quantum information systems.

## 2. Novel Mechanisms

SAW platforms are increasingly powered by physical mechanisms that extend far beyond classical piezoelectric actuation. Recent advances reveal that symmetry breaking in magnetoelastic and chemically anisotropic materials can introduce direction-dependent propagation and nonreciprocal diffraction, enabling new forms of wave control. At the same time, coherent optical access to SAWs realized through stimulated Brillouin processes allows photons to generate, probe, and engineer high-Q surface modes even in non-piezoelectric structures. Complementing these developments, acoustoelectric interactions in thin-film and hybrid substrates continue to provide powerful routes for tuning SAW velocity, charge transport, thermal fields, and environmental responses. These mechanisms mark a shift toward SAW devices governed by multiphysics interactions, offering unprecedented control over wave generation, modulation, and coupling across electromagnetic, optical, and mechanical domains.

### 2.1. Magnetoelastic Symmetry Breaking and Nonreciprocal Diffraction

**Figure 2:**
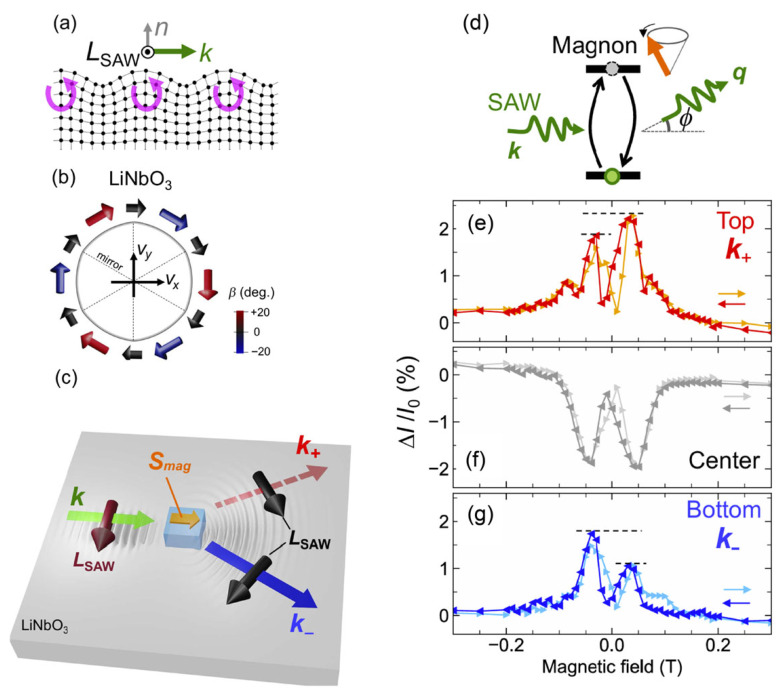
(**a**) Cross-section of a Rayleigh-type SAW showing the elliptical motion of lattice points and the associated SAW angular momentum $L_{SAW}$, oriented nearly along k×n, where k is the wavevector and n the surface normal. (**b**) Finite-element method (FEM) calculation of $L_{SAW}$ on the LiNbO_3_ Z-plane slowness curve; the arrow direction and color indicate the out-of-plane canting angle β. (**c**,**d**) Schematics of nonreciprocal SAW scattering via ferromagnetic resonance (FMR): an incident SAW excites a magnon with spin angular momentum $S_{mag}$, which re-emits SAWs at diffraction angles ± φ with unequal upward ($k_{+}$) and downward ($k_{-}$) amplitudes due to the chiral coupling between $L_{SAW}$ and $S_{mag}$. (**e**–**g**) Magnetic-field-dependent changes in SAW intensity measured at the top (*φ* = +30°, $k_{+}$), center (0°, transmission), and bottom (*φ* = −30°, $k_{-}$) IDTs; transmitted and diffracted signals are collected at the corresponding right-hand IDTs [141].

The nonreciprocal diffraction of SAWs provides a clear example of how magnetoelastic symmetry breaking can be engineered. As illustrated in Figure 2a, a Rayleigh-type SAW is confined near the surface of a solid and consists of coupled longitudinal and transverse motion, causing lattice points to follow elliptical trajectories [11,12]. In an isotropic medium, this ellipse lies in the plane defined by the surface normal n and the propagation direction k. This elliptical particle motion carries an intrinsic phonon angular momentum $L_{SAW}$ [13], which, to leading order, points along k×n. Figure 2b shows the finite-element-calculated distribution of $L_{SAW}$ on LiNbO_3_, where crystal anisotropy introduces a small out-of-plane canting angle β but preserves the dominant momentum–locked behavior $L_{SAW}\propto k\times n$. Such k-dependent angular momentum is analogous to spin–momentum locking in electronic surface states.

When a SAW encounters a ferromagnetic grating carrying spin angular momentum $S_{mag}$, the diffracted beams can become nonreciprocal. As summarized in Figure 2c, for $S_{mag}∥k$, the angular-momentum inner products satisfy $L_{SAW}\;\cdot \;S_{mag}<0$ for the upward ($k_{+}$) beam and $L_{SAW}\;\cdot \;S_{mag}>0$ for the downward ($k_{-}$) beam, producing inequivalent magnetoelastic coupling strengths. Figure 2d depicts the corresponding resonant scattering pathway: the incident SAW first excites a magnon in the ferromagnetic nanowire grating via magnetoelastic coupling, and this magnon subsequently re-emits SAWs into the ±φ diffracted orders. Because the scattering amplitude depends on the sign and magnitude of $L_{SAW}\;\cdot \;S_{mag}$, the upward and downward beams acquire different intensities, giving rise to nonreciprocal diffraction. Reversing $S_{mag}$ reverses the sign of $L_{SAW}\;\cdot \;S_{mag}$ and therefore reverses the diffraction asymmetry. Motivated by this symmetry argument, the experiment implements nonreciprocal SAW diffraction using a periodic array of magnetoelastic Ni nanowires as a SAW grating to enhance the diffracted orders [141].

The magnetic-field dependence of both the transmitted and diffracted SAWs is quantified using the microwave power transmittance $\left|S_{21}\right|$ between the left IDT and the right-side IDTs, averaged over 2.60–2.64 GHz after time-domain gating. All measurements are performed at 260 K in a split-coil superconducting magnet with a rotation stage, chosen for long-term thermal stability. When the magnetic field is applied along k, the transmission measured at the center detector (0°, Figure 2f) shows two nearly symmetric dips around ±50 mT. These features, widely reported in earlier works [142,143,144,145,146], are signatures of acoustically driven ferromagnetic resonance (acoustic-FMR), in which magnetoelastic coupling transfers energy from the SAW to the FMR mode when their frequencies coincide [147]. The symmetry of the dips indicates that directional nonreciprocity is weak for the k∥H configuration, consistent with previous observations [142,143,146].

In contrast, the diffracted beams exhibit clear nonreciprocity. As shown in Figure 2e for the upward $k_{+}$ beam (*φ* = +30°) and Figure 2g for the downward $k_{-}$ beam (*φ* = −30°), the relative intensity change $\Delta I/I_{0}$ shows pronounced ~2% peaks at the same magnetic fields as the transmission dips. These peaks reflect the resonant magnon-mediated scattering process depicted in Figure 2d. Most importantly, the peak heights depend on the sign of the magnetic field, and the asymmetry reverses when switching between the upward and downward diffraction directions, an unambiguous signature of nonreciprocal SAW diffraction. The magnitude and polarity of this asymmetry agree well with theoretical modeling of resonant SAW–magnon scattering, confirming the role of the $L_{SAW}\;\cdot \;S_{mag}$ interaction in determining the directional scattering contrast.

### 2.2. Coherent Optical Access to SAWs

**Figure 3:**
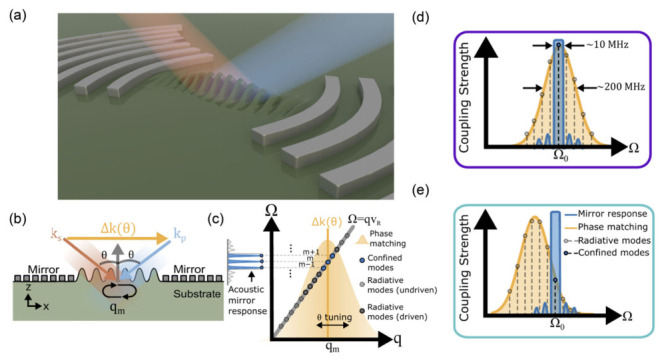
Parametric optomechanical interactions mediated by Gaussian SAW resonators. (**a**) Two non-collinear traveling optical beams are incident on a Fabry–Perot-type Gaussian SAW resonator. Their interaction is mediated by a Gaussian SAW cavity mode that is confined to the substrate surface. (**b**) Phase-matching diagram of the parametric process. The vector difference between the two optical wavevectors, Δk ≈ 2k_0_ sinθ, depends on the incidence angle θ and is directed along the SAW cavity axis. (**c**) Acoustic dispersion Ω(q) becomes discretized in the presence of the SAW cavity. The net optomechanical response arises only from modes that lie within both the optical phase-matching region and the acoustic mirror bandwidth (blue dots), whereas radiative longitudinal modes excluded by the mirror or phase-matching conditions (gray dots) do not contribute. (**d**) When the phase-matching envelope overlaps the mirror-defined mode, the optomechanical coupling is maximized. (**e**) Detuning between the phase-matching envelope and the mirror-selected SAW mode leads to a reduced optomechanical response [148].

The device consists of a Fabry–Perot Gaussian SAW resonator defined by two acoustic mirrors composed of periodically curved metallic reflectors on a single-crystal substrate. As illustrated in Figure 3a, two non-collinear optical beams (pump and Stokes) are incident into the cavity region. Their interference generates a traveling optical beat pattern that exerts a spatially periodic optical force capable of driving a confined Gaussian SAW cavity mode, provided that both energy and momentum conservation are satisfied. This configuration realizes a Brillouin-like parametric interaction that does not require piezoelectric coupling and therefore enables coherent optical access to SAW modes in virtually any crystalline material, including piezo-inactive substrates such as GaAs, Si, and diamond.

The phase-matching geometry is shown in Figure 3b. For pump and Stokes fields incident at equal and opposite angles ±*θ* relative to the surface normal, the in-plane optical wavevector mismatch is $\vec{\Delta k}\approx 2k_{0}\sin\theta$, directed along the acoustic cavity axis. The optical frequency difference Δω = ω_p_ − ω_s_ determines the target phonon frequency. Under ideal conditions, freely propagating Rayleigh SAWs satisfy $\Omega =qv_{R}$, so perfect phase matching selects $q_{0}=\Delta k$ and $\Omega_{0}=q_{0}v_{R}$.

Inside a cavity, however, the accessible phonon spectrum is strongly modified. As depicted in Figure 3c, the acoustic dispersion becomes discretized into standing-wave modes $q_{m}=m\pi /L_{eff}$ with corresponding frequencies $\Omega_{m}=q_{m}v_{R}$, where m is the longitudinal mode index and L_eff is the effective cavity length. Only a finite set of these modes falls within the acoustic mirror stop-band and is efficiently confined (blue circles). Modes that lie outside this mirror-defined bandwidth are radiative and do not contribute (gray circles).

The optomechanical coupling strength to each cavity mode is further shaped by the finite size of the optical beams. Because the Gaussian beams have a finite spatial extent, the optical forcing spectrum spans a range of wavevectors centered at Δ*k*. The single-phonon coupling rate therefore depends on the mismatch $\Delta k=q_{m}$ through a Gaussian envelope,

$$g_{0}(\Delta k)\propto exp[-(\Delta k-q_{m}{)}^{2}/\delta k^{2}],$$

where $\Delta k=2\sqrt{2}$/$r_{0}$ is set by the optical beam radius *r*_0_. Equivalently, the coupling as a function of incidence angle *θ* follows a Gaussian dependence centered at the phase-matching angle.

The resulting optomechanical spectrum thus consists of multiple discrete SAW cavity modes, but only those that lie both within the acoustic mirror bandwidth and within the optical phase-matching envelope contribute appreciably. Measurements of the Brillouin coupling coefficient as a function of *θ* confirm this behavior, showing a Gaussian angular dependence with a peak at *θ*_0_ ≈ 7.8° and a measured bandwidth of 0.9°, in excellent agreement with the predicted value of 0.8° for the experimental beam radius *r*_0_ ≈ 30 μm.

Strongest coupling occurs when the optical phase-matching envelope is centered on a mirror-selected cavity mode (Figure 3d). When the two are detuned (Figure 3e), the optomechanical response reduces accordingly. This tunability, achieved simply by varying the optical incidence angle, provides a powerful mechanism for engineering multi-mode or single-mode optomechanical gain spectra. It also enables precise spatial and spectral addressing of individual SAW modes, including higher-order transverse modes that are inaccessible to electromechanical drive. These results demonstrate that Gaussian SAW resonators support strong, tunable, and material-agnostic optomechanical coupling. This enables a versatile platform that unifies high-Q SAW confinement, angle-tunable Brillouin interactions, and optical-only access to phonons, offering new opportunities for SAW-based spectroscopy, sensing, and quantum hybrid systems.

**Figure 4:**
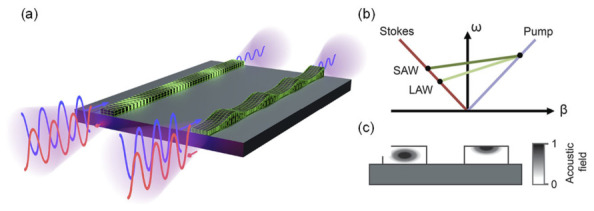
Concept of Brillouin backscattering from longitudinal and surface acoustic waves. (**a**) Schematic of on-chip Brillouin scattering driven by purely LAWs (left), depicted as density perturbations in the waveguide core, and by SAWs (right) propagating along the waveguide surface. (**b**) Optical dispersion diagram illustrating the Brillouin interaction with longitudinal and surface acoustic modes. The acoustic frequency (for both SAW and LAW) equals the frequency offset between the pump and Stokes waves. Because optical and acoustic velocities differ greatly, the diagram is intentionally exaggerated and not drawn to scale. (**c**) Representative acoustic displacement profiles of longitudinal and surface acoustic waves in the waveguide [149].

Figure 4 summarizes the physical basis of Brillouin backscattering from both bulk and surface-confined acoustic waves in integrated chalcogenide waveguides. In backward SBS, a strong optical pump at frequency $\omega_{p}$ interacts with a counter-propagating Stokes field at $\omega_{s}=\omega_{p}-\Omega_{B}$, generating an acoustic wave of frequency $\Omega_{B}$ through electrostriction and photoelasticity. This parametric interaction forms a closed feedback loop in which interference between pump and Stokes fields reinforces the acoustic wave, resulting in exponential amplification of the Stokes signal, a hallmark of stimulated Brillouin scattering.

Traditionally, backward SBS in integrated photonics couples predominantly to longitudinal acoustic waves (LAWs), whose displacement is confined within the waveguide core. This is illustrated in Figure 4a (left), where the elastic field resides in the high-index ${Ge}_{11.5}{As}_{24}{Se}_{64.5}$ core. Because LAWs propagate at the longitudinal acoustic velocity, the resulting Brillouin frequency shift is comparatively large. The SBS shift is given by:

$$\Omega_{B}=\frac{2n_{eff}v_{q}\;\omega_{p}}{c},$$

where $n_{eff}$ is the effective optical index, $v_{q}$ the acoustic phase velocity (longitudinal or surface), and c the speed of light. This expression, explains the strong sensitivity of $\Omega_{B}$ to geometry, strain, and temperature properties that underlie the utility of SBS for sensing applications [150,151,152].

In contrast, SAWs shown in Figure 4a (right) exhibit transverse, surface-bound displacement fields, as confirmed by the simulated mode profiles in Figure 4c. Because SAWs travel significantly more slowly than LAWs (typically 0.87–0.95 times the shear velocity), the associated Brillouin shift is correspondingly reduced. This distinction is emphasized in Figure 4b, where the LAW-mediated interaction yields a steeper optical dispersion slope (larger $\Omega_{B}$), whereas SAW-SBS produces a smaller pump–Stokes separation. The diagram is intentionally exaggerated, consistent with the original paper, because acoustic velocities are orders of magnitude lower than optical group velocities, making the true momentum mismatch too small to visualize.

The key contribution of the referenced work is the first experimental demonstration of on-chip SAW-mediated SBS, enabled by thin, high-index ${Ge}_{11.5}{As}_{24}{Se}_{64.5}$ waveguides engineered to maximize the overlap between optical modes and surface-localized acoustic fields. By optimizing the waveguide cross-section (≈2600 nm × 116 nm) and eliminating over-cladding layers that otherwise suppress surface motion, the authors achieve strong optical–SAW coupling. Measurements across an 8.5 cm on-chip circuit reveal a record SAW-SBS gain of 203 W^−1^ m^−1^ and a narrowing of the SAW resonance linewidth from 34 MHz to 20 MHz, consistent with stimulated amplification. Together, these results establish SAW-SBS as a new Brillouin interaction regime with promising implications for surface-sensitive sensing, tunable narrowband filtering, and integrated optomechanics.

### 2.3. Acoustoelectric/CMOS Control

**Figure 5:**
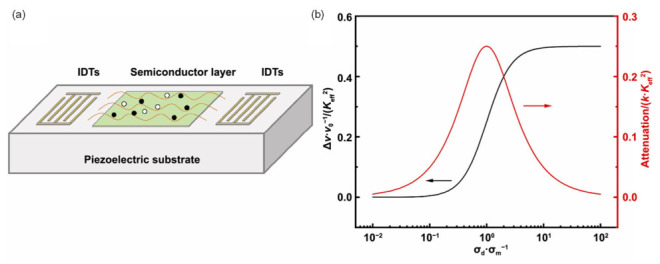
Concept and simulation results for the SAW devices. (**a**) Schematic illustration of the interaction between the surface acoustic wave and charge carriers. (**b**) Simulated phase velocity variation normalized by $K_{eff}^{2}$ and attenuation coefficient normalized by $k\cdot K_{eff}^{2}$ as functions of the conductivity ratio $\sigma_{d}/\sigma_{m}$ [156].

The acoustoelectric effect, i.e., the coupling between propagating piezoelectric SAWs and mobile charge carriers in an adjacent semiconductor, provides a powerful mechanism for electrically reconfigurable SAW devices. When a Rayleigh wave travels along a piezoelectric substrate, its mechanical deformation generates a periodic electric potential and accompanying near-surface electric fields. These fields interact with charge carriers in a thin conductive film placed on top of the substrate, modifying SAW propagation through two mechanisms: piezoelectric stiffening, which increases the phase velocity, and carrier-induced Ohmic loss, which introduces attenuation [153,154,155]. Figure 5a illustrates this interaction, where the SAW’s traveling electric fields modulate the carrier distribution in the semiconductor layer.

For a conductive layer much thinner than the SAW wavelength (d ≪ λ), the canonical perturbative model yields the normalized velocity shift and attenuation as [156,157].

$$\frac{\Delta v}{v_{0}}=\frac{K_{eff}^{2}}{2}\frac{1}{1+{\left(\sigma_{d}/\sigma_{m}\right)}^{2}}$$

$$\Gamma =k\frac{K_{eff}^{2}}{2}\frac{\left(\sigma_{d}/\sigma_{m}\right)}{1+{\left(\sigma_{d}/\sigma_{m}\right)}^{2}}$$

where $K_{eff}^{2}$ is the electromechanical coupling factor of the SAW, k=2π/λ is the wavevector, $\sigma_{d}$ is the sheet conductivity of the semiconductor film, and $\sigma_{m}=v_{0}\varepsilon_{0}(\varepsilon_{p}+\varepsilon_{s})$ is the relaxation conductivity at which SAW attenuation is maximized. The permittivities $\varepsilon_{p}$ and $\varepsilon_{s}$ correspond to the piezoelectric substrate and semiconductor overlayer, respectively.

Figure 5b plots the simulated dependence of normalized phase velocity shift and attenuation on the conductivity ratio $\sigma_{d}/\sigma_{m}$. The velocity decreases monotonically with increasing conductivity, approaching half the maximum possible shift for $\sigma_{d}=\sigma_{m}$. In contrast, attenuation exhibits a Lorentzian-like peak centered at $\sigma_{d}=\sigma_{m}$, where the SAW’s electric field drives the largest carrier current and, correspondingly, the greatest Ohmic dissipation. This characteristic behavior is a hallmark of acoustoelectric interaction and aligns well with prior reports on ZnO/GaN, GaAs/LiNbO_3_, and 2DEG-based devices.

Experimentally, the relative velocity shift can be extracted from the SAW phase change measured across a mesa of length $L_{mesa}$ via:

$$\frac{\Delta v}{v_{0}}=\frac{\lambda }{L_{mesa}}\frac{\Delta \phi }{360^{\circ }}.$$

This formalism is consistent with the analytical model and is used extensively in the design of voltage-tunable phase shifters. In the uploaded reference device (ZnO TFTs on LiNbO_3_), the achievable tuning range is ultimately governed by $K_{eff}^{2}$ of the substrate–mode combination, highlighting why high-$K^{2}$ substrates such as Y-cut LiNbO_3_ and higher-order modes (e.g., LLSAWs) yield substantially larger tunability than low-$K^{2}$ systems.

Finally, the original paper further corroborates these model predictions with 2D finite-element simulations (FEA) performed on 41° Y-cut LiNbO_3_, capturing variations in phase velocity, electromechanical coupling, and modal distribution across propagation angles. These simulations validate that higher-order modes such as the LLSAW exhibit significantly larger $K_{eff}^{2}$, explaining the enhanced tuning observed in fabricated devices.

## 3. Materials and Fabrication

Beyond conventional bulk piezoelectric wafers and subtractive lithography, recent progress in SAW technology has been driven by two parallel trends: the emergence of engineered 2D material platforms and the rise in additive, SAW-compatible fabrication strategies. Two-dimensional materials such as MXenes offer chemically addressable, atomically thin acoustic media, where surface terminations and layer stacking directly tune SAW velocity, confinement, and dispersion, enabling structurally programmable acoustic responses. In parallel, additive approaches ranging from aerosol jet printing of conductive interdigitated transducers to SAW-assisted vat-photopolymerization of magnetic composites are transforming how SAW devices and SAW-coupled materials are fabricated. Together, these developments point toward a generation of SAW systems in which the acoustic properties are co-designed with the electronic ink, the 2D film chemistry, and the printing physics, rather than being fixed by a single bulk piezoelectric substrate.

### 3.1. Two-Dimensional Materials and MXenes

To explore how MXene chemistry and stacking influence SAWs, atomistic simulations were carried out on Ti_3_C_2_T_x_ (T = O, F) using a ReaxFF force field [158,159,160] parameterized in [161]. This potential captures both the strong in-plane Ti–C bonding and the weaker van der Waals interactions between stacked layers, and has been benchmarked extensively for titanium carbide MXenes [161,162,163,164]. In ReaxFF, the total energy includes contributions from bond stretching and coordination penalties, lone-pair, angle and torsional terms, as well as long-range van der Waals and Coulomb interactions, enabling a unified description of intra- and interlayer physics.

**Figure 6:**
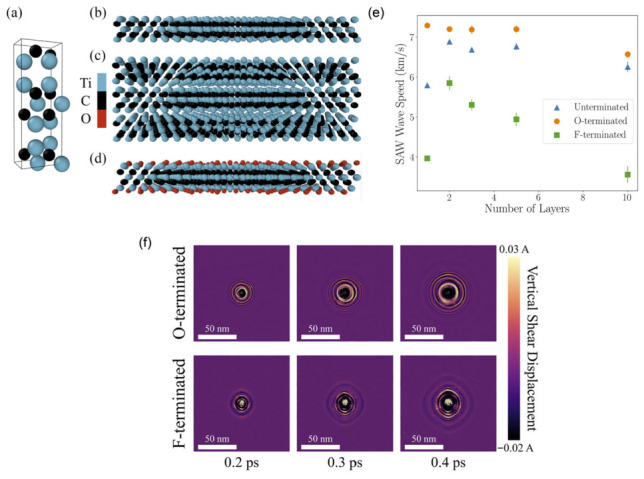
Surface acoustic waves in Ti_3_C_2_T_x_ MXene films: structure, stacking, and termination effects. (**a**) Primitive crystal structure of unterminated Ti_3_C_2_. (**b**) Isolated, unterminated Ti_3_C_2_ monolayer. (**c**) Three-layer unterminated Ti_3_C_2_ stack. (**d**) Single-layer Ti_3_C_2_ with O terminations. (**e**) Simulated SAW phase velocity as a function of MXene layer number for unterminated, O-terminated, and F-terminated films. (**f**) Snapshots of vertical shear displacement for two-layer O-terminated (top row) and F-terminated (bottom row) films at 0.2, 0.3, and 0.4 ps, plotted with identical spatial and color scales to highlight differences in wave propagation [165].

Initial configurations were constructed from the lattice parameters of pristine, and unterminated Ti_3_C_2_ [44], followed by the addition of O or F terminations and the assembly of multilayer stacks (Figure 6a–d). Each structure was first relaxed via conjugate-gradient minimization to optimize interlayer spacing, then equilibrated in an NPT ensemble at 100 K under zero in-plane stress. Subsequently, the systems were cooled to 10 K to suppress thermal noise in the displacement fields used for vibrational analysis. The simulated films span thicknesses from ≈5 Å for a single unterminated layer to ≈100 Å for a ten-layer O-terminated stack, with in-plane dimensions of ~140 nm × 140 nm to allow SAWs to propagate well beyond the initially perturbed central region. Representative structural parameters, including c-axis spacing and single-layer thicknesses (≈5 Å for pristine Ti_3_C_2_, 6.96 Å for O-terminated, and 8.35 Å for F-terminated layers), are summarized in the original work.

The resulting SAWs have low-THz frequencies and wavelengths on the order of 20–50 Å. For unterminated films, the simulated propagation velocities fall in the few-to-several km/s range, overlapping both reported SAW speeds in non-2D materials (3–8 km/s) and the higher values known for graphene (up to ~8–15 km/s, depending on mode and substrate). The finite simulation cell size and time window limit direct extraction of detailed dispersion relations, but the calculations nonetheless capture the essential features of SAW confinement, dispersion, and decay in ultrathin MXene films.

From the data in Figure 6e, three main trends emerge. First, there is a pronounced increase in SAW speed when going from one to two layers for pristine and F-terminated MXenes, whereas this jump is essentially absent for O-terminated films. This behavior mirrors the strong stiffening and increased bending rigidity observed experimentally when transitioning from monolayer to bilayer MXenes [166]. Second, surface terminations produce systematic shifts in velocity at fixed layer number: O-terminated structures tend to support faster SAWs than F-terminated ones, consistent with the higher elastic moduli and mechanical strength generally associated with oxygen termination [167,168]. Third, the time-resolved displacement maps in Figure 6f reveal qualitative differences in wavefront shape and amplitude between O- and F-terminated bilayers, reflecting termination-dependent phonon spectra and damping. Oxygen terminations introduce additional low- and mid-frequency vibrational modes [169], which modify the dynamic response and help explain the distinct SAW speeds and propagation patterns observed across pristine, O-terminated, and F-terminated MXene films. These simulations demonstrate that both layer number and surface termination are effective knobs for engineering SAW velocity and confinement in MXenes, positioning Ti_3_C_2_T_x_ as a tunable 2D platform for high-frequency acoustic and acousto-electronic devices.

### 3.2. Additive Electronics for SAW

**Figure 7:**
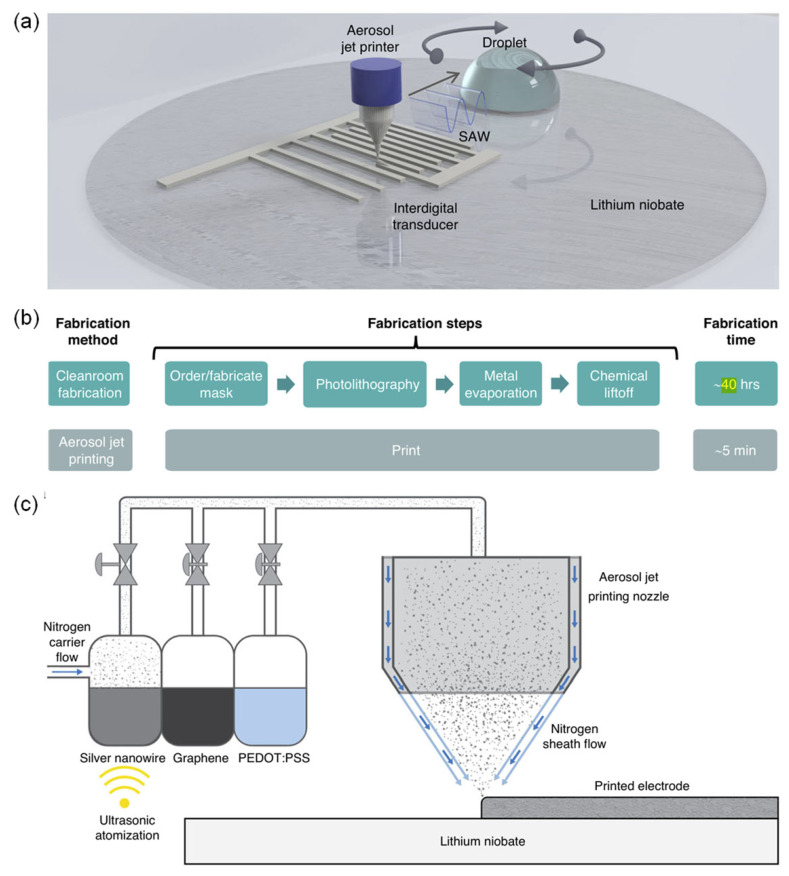
Aerosol jet printing of SAW microfluidic devices. (**a**) Schematic of an aerosol-jet-printed SAW microfluidic device. IDTs are directly patterned on a lithium niobate substrate and driven to generate SAWs that couple into an overlying droplet, where acoustic streaming and radiation forces manipulate the fluid and suspended particles. (**b**) Comparison of fabrication routes for SAW microfluidic devices. Conventional cleanroom processing requires mask fabrication, photolithography, metal deposition, and chemical lift-off, with a total turnaround of ~40 h, whereas aerosol jet printing completes the device in a single printing step in ~5 min. (**c**) Schematic of the aerosol jet printing workflow. Conductive inks (e.g., silver nanowire, graphene, PEDOT:PSS) are ultrasonically atomized, transported by a nitrogen carrier gas, focused by a coaxial nitrogen sheath flow inside a 150 μm nozzle, and deposited onto the lithium niobate substrate to form IDT electrodes [170].

As illustrated schematically in Figure 7a, IDT patterns are deposited directly onto the piezoelectric substrate using an aerosol jet printer (Optomec, Aerosol Jet technology, Albuquerque, NM, USA). Once printed, the IDTs can be driven immediately to launch SAWs, which couple into a sessile droplet and generate acoustic streaming and radiation forces. These forces enable typical SAW-microfluidic operations, such as rapid mixing, droplet actuation, and particle concentration, without any additional post-processing.

The benefit of this approach becomes clear in Figure 7b, which compares the overall process flow and turnaround time for conventional cleanroom fabrication versus aerosol jet printing. A standard lithographic route involves mask fabrication or ordering, photolithography, metal evaporation, and chemical lift-off, leading to a total processing time on the order of ~40 h and several discrete steps. In contrast, aerosol jet printing collapses the entire electrode-patterning sequence into a single printing step that can be completed in ~5 min, making it highly attractive for rapid prototyping, iterative design, and on-demand customization of SAW microfluidic devices.

The aerosol jet printing workflow is summarized in Figure 7c. A chosen conductive ink (such as silver nanowire, graphene, or PEDOT:PSS) is first atomized by an ultrasonic transducer to create a fine aerosol. This aerosol is transported toward the print head by a nitrogen carrier gas, then hydrodynamically focused by a concentric nitrogen sheath flow as it passes through a 150 μm nozzle. The resulting narrow, well-collimated jet enables precise deposition of IDT fingers with controlled line width and spacing directly on lithium niobate, while remaining compatible with a wide variety of functional inks and substrate materials.

**Figure 8:**
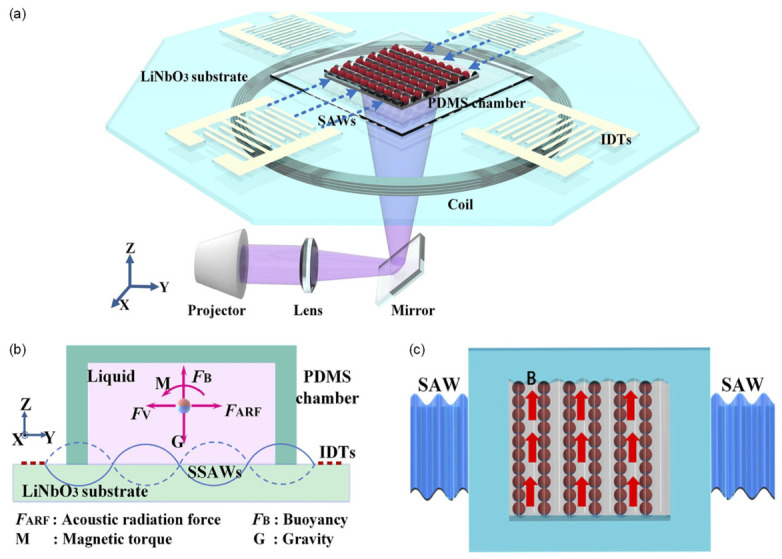
SAW-assisted printing of magnetic composites. (**a**) Schematic of the integrated fabrication platform combining a SAW device, a PDMS chamber containing magnetic-resin mixtures, an external electromagnetic coil, and a DLP projector for patterned UV curing. (**b**) Force diagram for a magnetic particle suspended in the liquid resin, illustrating the contributions of acoustic radiation force $F_{ARF}$, viscous drag $F_{V}$, gravitational force G, buoyancy $F_{B}$, and magnetic torque M under the standing SAW field. (**c**) Alignment of Fe_3_O_4_ particles under a directional magnetic field, where magnetic dipoles rotate to align along the applied field direction [171].

The SAW-assisted printing system used to pattern magnetic composites is illustrated in Figure 8a. The setup integrates a SAW device, a polydimethylsiloxane (PDMS) chamber for holding the magnetic-resin suspension, an electromagnetic excitation coil, and a high-resolution digital light processing (DLP) projector for spatially selective photopolymerization. The SAW device is fabricated on a 128° Y-cut LiNbO_3_ substrate using patterned IDTs, which generate Rayleigh-type SAWs upon electrical excitation. Two orthogonal pairs of IDTs are arranged at 45° relative to the X-axis of the substrate, ensuring the generation of stable SAWs with identical resonant frequency and amplitude. Each IDT consists of 20 electrode pairs with a finger width of 100 μm, giving a pitch of 200 μm. Because the acoustic wavelength is four times the finger width, the resulting pressure nodes/antinodes form particle trapping planes spaced at twice the finger width. For the given geometry and Rayleigh velocity of LiNbO_3_, the operating frequency is approximately 9.4 MHz, consistent with the original report.

Device fabrication follows standard UV lithography on LiNbO_3_, after which a 200 nm-thick Al layer is deposited by magnetron sputtering [172]. The fabricated device demonstrates a mechanical quality factor of ~62.7 and an acoustic velocity of ~3760 m/s, and maintains stable performance over years of storage. Under repeated use in resin environments, however, IDT degradation typically limits operational lifetime to around three months.

During operation, the signal generator (Tektronix AFG3052C, Shanghai, China) drives the IDTs, and the amplified RF output launches SAWs into the PDMS chamber situated above the IDT region. The chamber contains a photocurable resin uniformly mixed with Fe_3_O_4_ particles. As SAWs propagate beneath the chamber, they generate a spatially periodic acoustic pressure field that redistributes the suspended particles into well-defined bands. An electromagnetic coil wrapped around the chamber supplies an external magnetic field oriented approximately along the z-axis, which imposes a directional magnetic torque on the particles.

The force balance acting on an individual particle is summarized in Figure 8b. The acoustic radiation force $F_{ARF}$ drives particles toward nodes or antinodes of the standing SAW field, while viscous drag $F_{V}$ opposes motion. Gravity G and buoyancy $F_{B}$ act along the vertical direction, and an applied magnetic field produces a torque M that rotates the magnetic dipole moments of the Fe_3_O_4_ particles. After the SAW-induced pattern becomes stable, a 200 mHz, 1.0 V square-wave signal is applied to the coil to generate a time-varying directional magnetic field, causing the particles to align along the field direction, as shown in Figure 8c. Finally, the DLP projector illuminates the desired pattern, curing the resin and fixing the magnetically aligned structures in place. This hybrid acoustic–magnetic–photonic approach enables high-resolution patterning of magnetic composites with tunable particle orientation and programmable spatial architectures, offering a versatile route toward soft robotic elements, functional metamaterials, and micro-actuated devices.

## 4. Devices and Architectures

Rapid advances in device architectures are reshaping the landscape of SAW technology, enabling functionalities that extend far beyond classical fixed-frequency filters and delay lines. Two developments in particular electrically reconfigurable phase shifters and ultra-high-frequency delay-line sensors highlight how innovations in materials, nanoscale transduction geometries, and acoustoelectric coupling are producing SAW platforms with unprecedented tunability, compactness, and sensitivity. Recent work demonstrates that thin-film transistor–integrated SAW structures can deliver voltage-controlled phase modulation with high efficiency, while GHz-scale delay-line sensors leverage sub-micron IDTs and engineered propagation paths to realize temperature- and environment-sensitive devices suitable for modern wireless and embedded sensing systems.

### 4.1. Reconfigurable Phase Shifters

**Figure 9:**
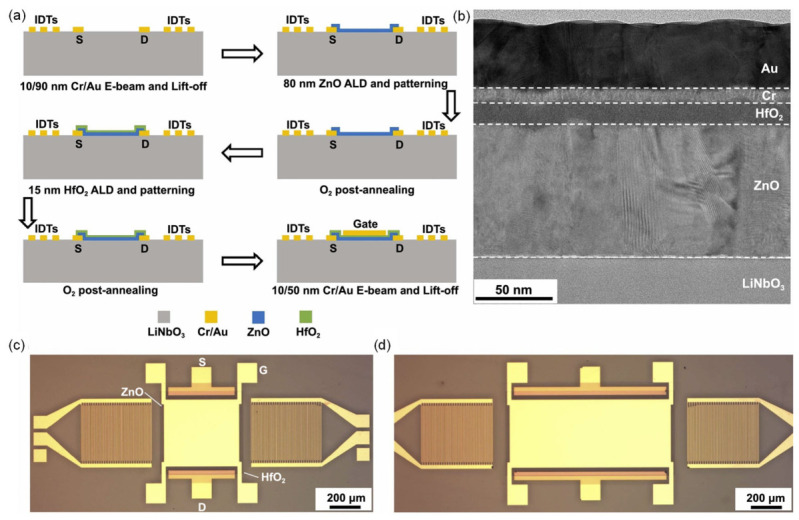
Fabrication and structural characterization of ZnO/HfO_2_/LiNbO_3_ acoustoelectric SAW phase shifters. (**a**) Process flow for devices with mesa lengths of 250, 500, 750, 1000, 1250, and 1500 µm. Cr/Au IDTs and source–drain contacts are first defined on 41° Y-cut LiNbO_3_, followed by ALD deposition and patterning of the ZnO channel, HfO_2_ gate dielectric, and Cr/Au gate electrodes. (**b**) Cross-sectional STEM image of the completed TFT stack, showing (from bottom to top) the LiNbO_3_ substrate, ZnO semiconductor, HfO_2_ dielectric, and Cr/Au gate metal. (**c**) Optical micrograph of a device with a mesa length of 500 µm. (**d**) Optical micrograph of a device with a mesa length of 1000 µm [156].

The fabrication flow for the acoustoelectric SAW phase shifters with mesa lengths of 250, 500, 750, 1000, 1250, and 1500 µm is summarized in Figure 9a. First, 10/90 nm Cr/Au electrodes are patterned on 41° Y-cut LiNbO_3_ by electron-beam evaporation and lift-off. These metallizations define both the IDTs used to launch and detect SAWs and the source/drain contacts of the ZnO thin-film transistor (TFT). The IDT finger width and spacing are each 3.5 µm, and the acoustic aperture is set to 350 µm, slightly smaller than the 400 µm mesa width to ensure that the propagating SAW is fully confined within the electrically modulated region.

An 80 nm ZnO semiconductor layer is then deposited by ALD at 200 °C using diethylzinc and deionized water as precursors, and patterned by 6:1 buffered oxide etch (BOE) to define the active channel. The devices undergo an O_2_ anneal at 300 °C for 30 min to suppress the intrinsic conductivity of the as-grown ZnO. Next, a 15 nm HfO_2_ gate dielectric is deposited by ALD at 250 °C using TDMAHf and H_2_O, patterned by Cl_2_/BCl_3_ ICP etching, and subjected to a second O_2_ anneal at 300 °C for 30 min to further improve the interface quality. Finally, 10/50 nm Cr/Au gate electrodes are deposited and lifted off, completing the ZnO/HfO_2_ TFT stack. A representative cross-sectional STEM image is shown in Figure 9b, while Figure 9c and Figure 9d present optical micrographs of devices with 500 µm and 1000 µm mesa lengths, respectively.

Because the phase shifter performance relies on modulating SAW propagation via the carrier density in the ZnO film, controlling the ZnO conductivity is crucial. ALD enables highly uniform ZnO at relatively low temperatures, but the resistivity is strongly affected by growth and annealing conditions. Higher deposition temperatures and oxygen-poor environments enhance the incorporation of hydrogen donors and promote oxygen vacancies, both of which can increase the free-carrier density through shallow donor complexes. Conversely, high-temperature annealing in O_2_ heals vacancy-related defects and drives out hydrogen from the near-surface region, thereby reducing the electron concentration. The O_2_ anneals incorporated into the flow of Figure 9a are therefore essential steps for tuning the ZnO into a moderately conducting regime where the acoustoelectric effect yields large, yet controllable, SAW phase shifts.

### 4.2. UHF Delay-Line Sensors

**Figure 10:**
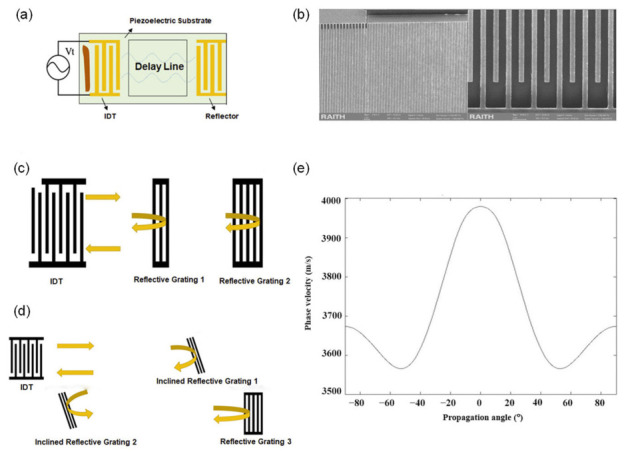
Ultra-high-frequency (UHF) SAW delay-line devices using nanoscale IDTs. (**a**) Schematic of a single-port SAW delay-line structure consisting of an IDT and reflective gratings fabricated on a piezoelectric substrate. (**b**) SEM images of the fabricated 350 nm–scale IDTs produced by high-resolution electron-beam lithography. (**c**) Conventional parallel delay-line architecture using two short-circuit reflector gratings. (**d**) Z-shaped delay-line configuration employing inclined reflectors to increase delay without expanding lateral footprint. (**e**) Direction-dependent Rayleigh phase velocity of 128° YX LiNbO_3_ as a function of propagation angle, used to determine practical reflection angles for Z-path designs [174].

The SAW delay-line sensor used in this work consists of a single IDT and a sequence of reflective gratings patterned on a piezoelectric substrate, as shown schematically in Figure 10a. In a single-port configuration, the IDT converts the input RF excitation into a SAW, which propagates along the substrate surface, reflects from the gratings, and returns to the same IDT. This round-trip propagation introduces a well-defined time delay in the electrical response, enabling sensing by monitoring changes in delay time or the corresponding phase shift [173].

To realize gigahertz-range operation, the delay-line is fabricated on a 128° YX LiNbO_3_ substrate, where the Rayleigh velocity is $v_{s}\approx 3965\;m/s$. The resonant frequency is given by $f=v_{s}/\lambda$, so achieving UHF operation requires sub-micrometer wavelengths. In this design, a Rayleigh wavelength of 1.4 μm was selected, setting both the IDT finger width and center-to-center spacing to λ/4=350 nm. These nanoscale features were patterned using high-resolution electron-beam lithography with a double-layer PMMA resist stack and a conductive discharge layer to prevent charge accumulation during exposure. This process suppresses pattern collapse during development and enables reliable lift-off of narrow metal fingers. SEM images of the resulting nanoscale IDTs are shown in Figure 10b.

Devices with 20, 40, and 60 electrode pairs were fabricated, together with sensors having IDT apertures of 70, 85, and 100 μm. Before patterning the reflectors via e-beam exposure, their placement must be specified since the one-way distance l between the IDT and the first reflector directly sets the total delay $\tau =2l/v_{s}$. The corresponding phase shift [18] is

ϕ=2πf τ.

Any perturbation that alters the SAW velocity, for example, temperature, stress, mass loading, or fluid interaction, modifies τ and ϕ, enabling high-sensitivity detection. A conventional parallel delay-line configuration is shown in Figure 10c, where two short-circuit reflector gratings are placed parallel to the IDT. In the demonstrated device, the first reflector is located 2010 μm from the IDT, and the second at 2610 μm. This arrangement increases the total delay while maintaining simple fabrication.

To further enhance delay without proportionally enlarging the lateral footprint, a Z-shaped delay line was designed, illustrated in Figure 10d. In this configuration, the SAW first travels 2010 μm to a tilted open-circuit reflector inclined by 10.5°. Upon reflection, the wave is redirected by 21°, then propagates another 1150 μm to a second inclined reflector. A subsequent reflection guides the wave along a final 600 μm path toward a third short-circuit reflector. These angles were selected based on the directional Rayleigh-velocity plot for 128° YX LiNbO_3_ in Figure 10e, which shows significant velocity anisotropy with propagation direction.

The reflection angles cannot be chosen arbitrarily. If too small, the reflected wavefronts re-enter the IDT aperture and interfere with the outgoing SAWs. If too large, the SAW propagates along directions with substantially lower phase velocity, causing overlapping wave packets and degraded time resolution. The chosen angles therefore represent an optimized trade-off between maximizing delay, maintaining adequate temporal separation of reflections, and avoiding distortion due to anisotropic wave propagation. These UHF delay-line sensors demonstrate how nanoscale lithography combined with angle-engineered reflector geometries can achieve compact, high-sensitivity SAW devices suitable for next-generation timing, signal processing, and sensing applications.

## 5. Applications and Systems

Beyond materials and device architecture, SAW technology is increasingly shaping complete functional systems across sensing, microfluidics, thermal control, and soft robotics. Recent advances highlight how SAWs can serve simultaneously as actuators, energy transfer mechanisms, and sensing elements, enabling wireless defogging and de-icing platforms on glass, high-efficiency atomizers for inhalation therapy, fully printed microfluidic processors, and acoustically patterned magnetic soft-robotic components. Together, these emerging applications demonstrate the versatility of SAW-driven systems and mark a transition from isolated devices to multifunctional, integrated platforms capable of interacting with their environment in complex and programmable ways.

### 5.1. Wireless Defog/De-Ice + Sensing on Glass

**Figure 11:**
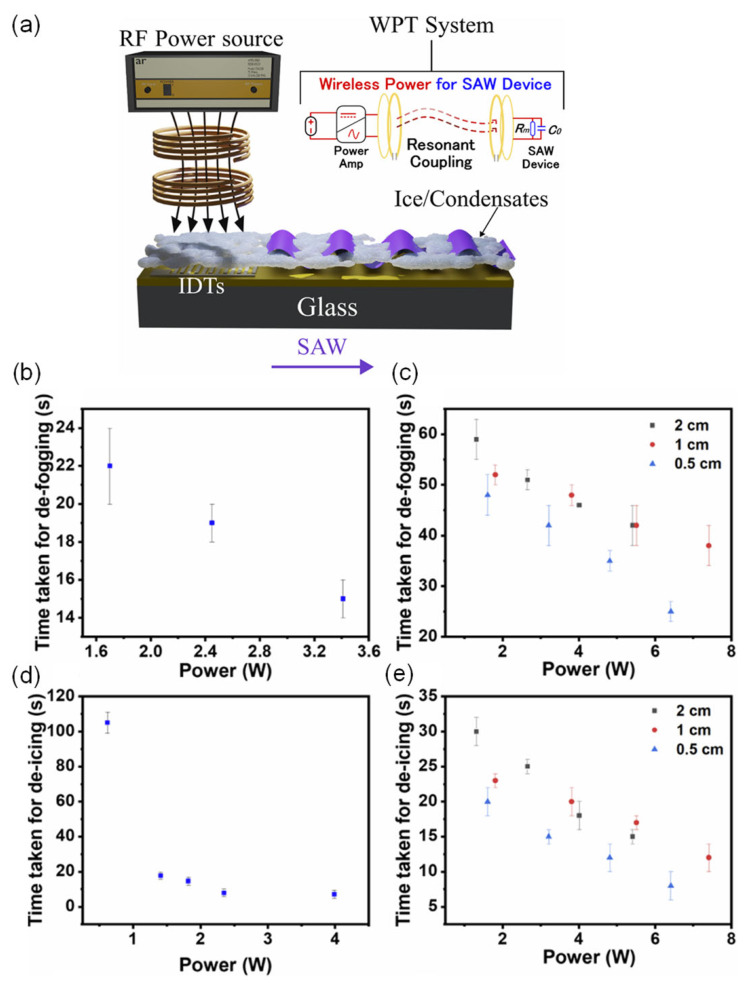
Wireless-powered SAW platform for defogging and de-icing. (**a**) Schematic of a ZnO/glass SAW device powered through an inductive wireless power transfer (WPT) link. An RF source drives the transmitting coil; resonant coupling delivers RF power to the receiving coil connected to the SAW IDTs, and the launched SAWs remove condensate/ice from the glass surface. (**b**) Defogging time of the wired (directly driven) SAW device as a function of RF power. (**c**) Defogging time of the wireless system versus RF power for different coil separations (0.5, 1, and 2 cm). (**d**) Rime-ice de-icing time of the wired system as a function of RF power. (**e**) De-icing time of the wireless system versus RF power and coil separation [175].

In this work, a wireless power transfer (WPT) system is co-designed with a ZnO/glass SAW device such that the WPT resonant frequency matches the acoustic resonance, enabling efficient wireless excitation at the device’s operating frequency. As sketched in Figure 11a, an RF power source and amplifier drive a transmitting coil. Through resonant inductive coupling, RF power is delivered to a receiving coil connected to the SAW IDTs, which launch Rayleigh waves along the glass substrate. The result is a thin-film SAW + WPT platform capable of surface sensing, defogging, and de-icing. For sensing, shifts in the SAW resonance frequency are monitored under low-temperature conditions, where temperature changes, electrical loading, and mass loading from condensates or ice all perturb the acoustic velocity. Here we focus on the active functionalities defogging and de-icing and compare wired (direct drive) and wireless operation of a 9.88 MHz ZnO/glass SAW device.

The defogging performance is summarized in Figure 11b,c. In the wired configuration (Figure 11b), the defogging time decreases monotonically with increasing RF power. This behavior reflects SAW-induced mechanical vibration and acousto-thermal effects in the ZnO/glass stack: propagating waves dislodge and coalesce condensate droplets and locally heat the surface, promoting evaporation and clearing of the fogged region. The wireless system (Figure 11c) shows the same qualitative trend: higher RF power leads to shorter defogging times but the absolute times are longer and the required power higher, owing to losses in the WPT link. Varying the separation between the transmitter and receiver coils from 0.5 to 2.0 cm reveals that shorter coil distances yield faster defogging at a given power level, consistent with improved coupling efficiency.

The de-icing response for rime ice is presented in Figure 11d,e. For the wired SAW device (Figure 11d), de-icing time again falls as RF power increases. The intense acoustic field at the rime-ice/glass interface produces strong interfacial shear and normal stresses that fracture and compact the porous ice layer, while acousto-thermal dissipation in the ZnO film and interfaces generates localized heating. Together, mechanical agitation and interfacial heating cause shrinkage, melting, and eventual detachment of the rime ice. In the wireless configuration (Figure 11e), de-icing times decrease with both increasing RF power and decreasing coil separation, mirroring the defogging trends. The WPT efficiency peaks around a coil spacing of ~1 cm, but in practice the shortest times are obtained at ~0.5 cm, where the combined effects of efficient power transfer and strong SAW-induced heating maximize de-icing efficiency.

Finally, it is important to note that coupling between the WPT resonators and the SAW load can lead to frequency splitting in the wireless link, shifting the effective resonance of the coupled system. If this shifted resonance is not tracked, the WPT impedance increases and the transfer efficiency drops, necessitating higher input power to achieve the same defogging or de-icing performance. This highlights the need for co-design of the WPT circuitry and SAW device in future wireless SAW anti-icing systems.

### 5.2. SAW Atomization for Inhalation

**Figure 12:**
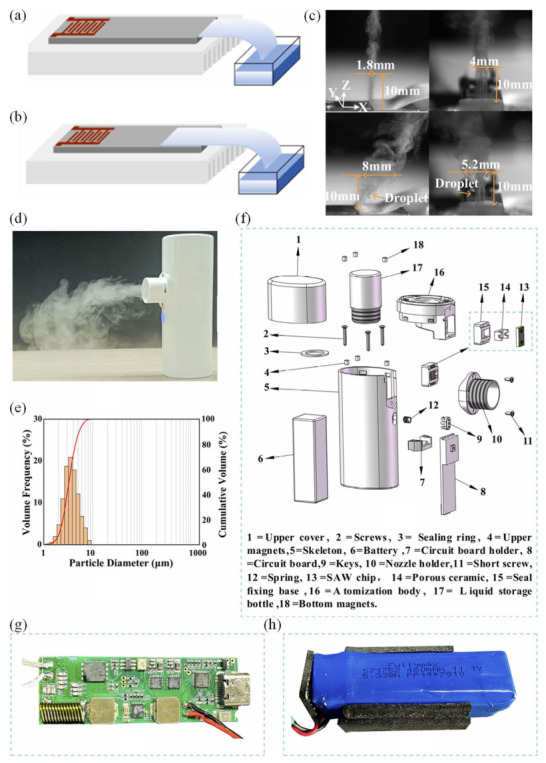
SAW atomization using the PSLEA (paper-strip liquid edge atomization) method. (**a**) Schematic of PSLEA mode, where a capillary wick supplies liquid to the edge of the SAW chip, creating a short liquid film. (**b**) Schematic of the conventional PSLSA (paper-strip liquid surface atomization) mode, where the strip lays across the chip surface and forms a long liquid film. (**c**) Optical images of aerosol plumes generated at 800 mV for both configurations, showing improved collimation and reduced droplet splash in the PSLEA mode. (**d**) Photograph of the handheld SAW atomizer implementing PSLEA. (**e**) Particle-size distribution of the generated aerosol from deionized water. (**f**) Exploded structural diagram of the handheld atomizer. (**g**) Driving circuit board. (**h**) Rechargeable battery module [176].

To supply liquid continuously to the atomization region, a polyester–cellulose fiber strip was used as a capillary wick. One end of the strip remained immersed in the liquid reservoir, while the opposite end contacted the SAW chip. In the PSLEA configuration shown in Figure 12a, the wick touches only the edge of the atomizer surface, forming a short liquid film directly at the SAW interaction zone. In contrast, the PSLSA configuration (Figure 12b) positions the wick across the entire atomizer surface, generating a much longer liquid film. These two modes were systematically compared to evaluate atomization performance.

The key distinction between PSLEA and PSLSA lies in the effective acoustic interaction length. In PSLSA, the long liquid film attenuates a significant portion of the acoustic energy as the SAW propagates through it, creating irregular capillary-wave patterns and enabling intermittent ejection of large droplets. The PSLEA mode minimizes this interaction length, reducing viscous damping and concentrating acoustic energy at the liquid edge. As a result, more SAW energy reaches the air–liquid interface, producing stronger capillary-wave excitation and superior aerosolization.

The atomization behavior at 800 mV for both methods is shown in Figure 12c. In PSLEA mode, a stable and highly directional aerosol plume forms, with a cross-section of ~1.8 mm × 4 mm at 10 mm above the chip. In PSLSA, the plume broadens to ~8 mm × 5.2 mm and contains both fine mist and large droplets, with significant angular deviation from the chip normal. This confirms that PSLEA generates a more collimated, uniform, and consistent aerosol jet.

Excessively long or unstable liquid films tend to produce fluctuating surface profiles, leading to droplet-size irregularity and reduced atomization quality. In contrast, the short, well-defined liquid edge in PSLEA supports regular capillary-wave formation and promotes finer aerosolization. These advantages translate directly into better spray quality, narrower particle-size distributions, higher deposition efficiency in the lungs, and reduced waste of liquid drug formulations.

A portable handheld SAW atomizer based on the PSLEA method is shown in Figure 12d. Because atomization is driven purely by acoustic waves without moving parts or an air jet, the device is compact, quiet, and suitable for infant and elderly patients. The aerosol particle-size distribution obtained from deionized water (Figure 12e) is unimodal with a median diameter of ~3.95 μm, ideal for pulmonary drug delivery to deep lung regions.

The internal structure of the handheld device (Figure 12f) comprises an atomization cup housing the SAW chip, a porous wick, a liquid reservoir, and sealing components. The power unit includes a dedicated SAW driving circuit (Figure 12g) and a rechargeable battery (Figure 12h). During operation, the wick delivers liquid to the chip edge, where SAWs atomize it continuously. Long-duration tests demonstrated stable plume formation even as the reservoir level dropped, indicating efficient and reliable capillary feeding and acoustic atomization.

### 5.3. Printed SAW Microfluidics

**Figure 13:**
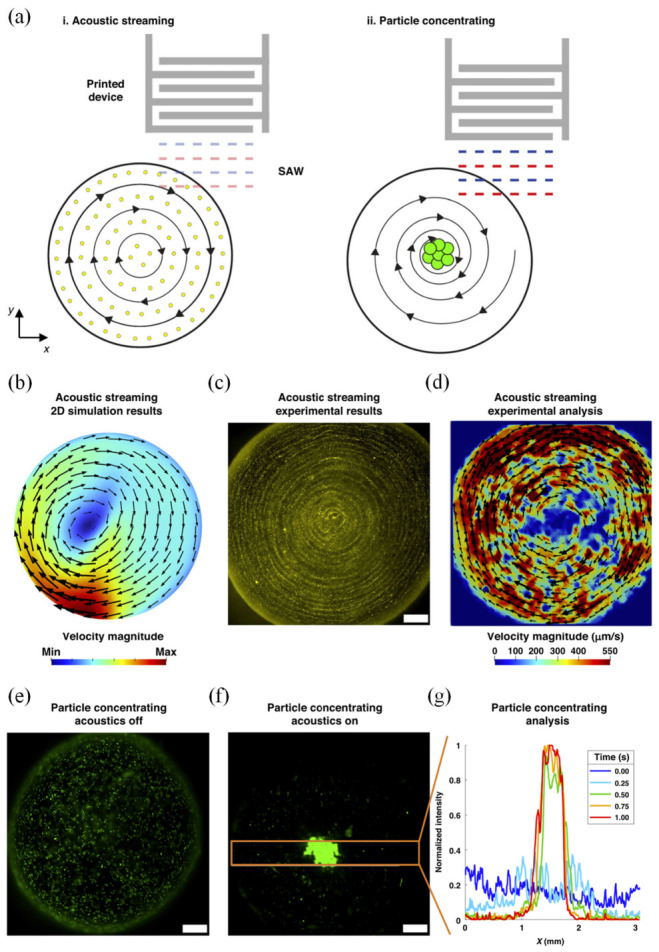
Microfluidic performance of aerosol-jet-printed SAW devices. (**a**) Schematics of two droplet-scale operations: (**i**) acoustic streaming and (**ii**) particle concentration, with relative SAW amplitude indicated by the color/opacity of the incident wave. (**b**) 2D simulation of streaming velocity magnitude and flow field inside a droplet excited by a traveling SAW incident from one side. (**c**) Experimental image of 2 μm tracer particles streaming in a 5 μL droplet driven by a silver-nanowire SAW device at 65.2 $V_{pp}$. (**d**) Particle-image-velocimetry (PIV) map corresponding to (**c**), showing velocity vectors and magnitude. (**e**–**g**) Concentration of 10 μm particles in a 5 μL droplet using the same device at 88.8 $V_{pp}$: (**e**) initial particle distribution with acoustics off; (**f**) distribution 1 s after SAW activation; (**g**) normalized fluorescence intensity in the region of interest indicated by the orange box in (**f**), plotted over the first 1 s of actuation. All scale bars: 400 μm [170].

To demonstrate the functionality of aerosol-jet-printed SAW devices in droplet microfluidics, acoustic streaming and in-droplet particle concentration were investigated, as summarized in Figure 13. Conceptually, when a slightly off-center sessile droplet is excited by a traveling SAW, both acoustic radiation forces and streaming flows act within the droplet. By adjusting the applied power and considering the particle size, the same platform can be operated either in a streaming-dominated regime or in a regime that produces strong particle focusing (Figure 13a).

The simulated streaming field in Figure 13b shows the velocity magnitude and flow pattern in a 5 μL droplet driven by an incident SAW from one side. The vortex center is displaced from the geometric center of the droplet, reflecting the asymmetry of the traveling wave–droplet interaction. Full 3D simulations of streaming and particle trajectories, discussed in the original work, confirm the formation of a single, large-scale recirculating flow.

Experimentally, acoustic streaming is visualized in Figure 13c using 2 μm tracer particles and a silver-nanowire SAW device with 100 μm finger width, driven at 65.2 $V_{pp}$. The corresponding PIV analysis in Figure 13d yields average streaming speeds on the order of 550 μm/s, in good agreement with the simulated profile in Figure 13b. These results verify that aerosol-jet-printed IDTs can generate strong, well-defined streaming flows suitable for mixing and transport in microliter droplets.

To showcase particle manipulation, the same device was used to concentrate 10 μm particles in a 5 μL droplet (Figure 13e–g). With acoustics off, particles are nearly uniformly distributed (Figure 13e). One second after SAW activation at a higher drive level of 88.8 $V_{pp}$, particles begin to migrate and accumulate near the droplet center (Figure 13f), indicating the onset of strong acoustic forces. The time evolution of concentration is quantified in Figure 13g, which plots the normalized fluorescence intensity along the horizontal axis within the highlighted region. The progressive growth of the central intensity peak over the first second reflects rapid particle focusing.

The underlying concentration mechanism, studied extensively in prior work [78,79,80,81,82,177], arises from the combined action of acoustic streaming, acoustic radiation forces, and secondary flows (including centrifugal effects) generated by the interaction of traveling and partially reflected SAWs in the droplet. The focusing efficiency depends on multiple parameters, SAW frequency and power, attenuation length in the liquid, droplet size and contact angle, and particle size and density, but the results in Figure 13 demonstrate that aerosol-jet-printed SAW devices can reliably deliver both high-speed streaming and robust, time-resolved particle concentration using simple printed electrodes.

### 5.4. Magnetic Soft-Robotic Components

**Figure 14:**
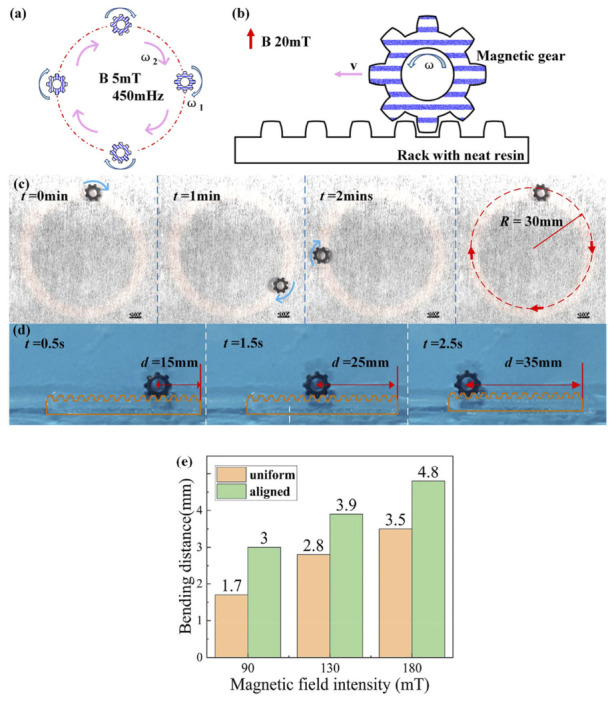
Magnetically actuated motion and gear–rack transmission of SAW-printed magnetic composites. (**a**) Schematic of a magnetic gear exhibiting simultaneous self-rotation and orbital motion under a rotating magnetic field. (**b**) Schematic of a printed magnetic gear engaging with a rack, converting rotation into linear displacement. (**c**) Experimental images showing gear rotation along a circular trajectory of radius ~30 mm. (**d**) Sequential snapshots of gear–rack meshing, demonstrating conversion of rotational motion into translational motion. (**e**) Measured bending displacement of uniform and magnetically aligned cantilever beams under magnetic fields of 90–180 mT [171].

Beyond simple self-rotation, the SAW-assisted-printed magnetic gear exhibits a variety of magnetically driven motion modes, demonstrating that the printing method can produce functional magnetic components capable of torque transmission, guided motion, and noncontact actuation. Here, two characteristic modes, circular motion and gear–rack engagement, highlight the versatility of the printed magnetic structures. As illustrated in Figure 14a, the magnetic gear is placed above a circular excitation coil with a diameter of 60 mm. When the coil is driven at 450 mHz and 3.5 V, it generates a rotating magnetic field of approximately 5 mT. This field induces simultaneous self-rotation of the gear and orbital motion along the coil path. Experimental motion sequences in Figure 14c show the gear completing one revolution along a circular trajectory of radius ~30 mm. The translational velocity is ~1.12 mm/s, while the angular velocity is ~2.1 rad/s. These two motions occur independently but are both governed by the amplitude and frequency of the applied magnetic field.

The mechanism arises from the magnetically aligned Fe_3_O_4_ particles within the gear. During SAW-assisted printing, the Fe_3_O_4_ particles align such that their dipole moments point vertically upward. When the gear is positioned above the excitation coil, its out-of-plane magnetization interacts with the radially directed component of the coil’s magnetic field. This interaction generates a torque driving self-rotation. Meanwhile, magnetic field gradients along the radial direction create potential wells, confining the gear to the annular region and producing the tangential force necessary for continuous orbital motion.

A second actuation mode, gear–rack, engagement is shown schematically in Figure 14b. Here, a magnetically aligned gear is placed in contact with a printed magnetic rack of matching tooth geometry. When exposed to an external magnetic field, the gear rotates counterclockwise, and the meshing interaction converts this rotation into linear displacement along the rack. Sequential snapshots in Figure 14d illustrate this conversion, showing the gear moving from right to left with an average speed of ~6.25 mm/s. The motion is stable and repeatable, demonstrating effective mechanical transmission through fully printed magnetic structures.

The enhanced performance of these structures arises from the magnetic alignment achieved during SAW-assisted printing. As quantified in Figure 14e, cantilever beams printed with aligned Fe_3_O_4_ particles exhibit significantly larger bending displacements than uniformly mixed composites under identical magnetic fields (90–180 mT). This increased responsiveness confirms that directional particle alignment improves magnetic torque generation and overall actuation efficiency. These results demonstrate that SAW-assisted printing enables fabrication of high-performance magnetic composites capable of complex, programmable motion under external magnetic fields. Such capabilities highlight the potential of this technique for developing soft robotic components, micro-actuators, and wirelessly controlled mechanical systems.

A comprehensive comparison table (Table 1) merging the technological mechanisms, material specifications, applications, and key performance metrics is presented below.

## 6. Cross-Cutting Challenges and Future Outlook in Surface Acoustic Wave Technology

The relentless push of SAW devices into UHF regimes to meet the demands of applications such as 5G communications and advanced sensing is exposing fundamental physical limitations where energy dissipation and thermal instability become the dominant barriers to performance and reliability. A critical hurdle in this high-frequency domain is maintaining a high quality factor and low energy loss, a multifaceted problem stemming from the device’s constituent materials and structures. The metallization of IDTs, for instance, introduces a mass-loading effect that inherently lowers the operational frequency; this issue is exacerbated by denser materials like platinum and thicker films, such as gold layers exceeding 50 nm.

Concurrently, ohmic losses within these metallic reflectors act as another significant channel for energy dissipation, particularly in piezo-active device configurations. However, the ultimate performance ceiling is most profoundly dictated by the piezoelectric substrate itself, where the choice of crystalline cut such as 128° YX LiNbO_3_, 41° Y-cut LiNbO_3_, or [100]-cut GaAs is the critical determining factor for both the strength of electromechanical coupling (K^2^) and the nature of the accessible acoustic modes, from Rayleigh to Longitudinal Leaky SAW (LLSAW). Even in an idealized structure, intrinsic phonon dissipation through propagation and mirror losses imposes a ceiling on performance. In certain GaAs cavities at cryogenic temperatures, mirror losses have been shown to dominate, whereas at higher temperatures, intrinsic propagation losses become the primary limiting factor.

Despite these inherent loss mechanisms, remarkable progress has been demonstrated, with optically coupled GaAs cavities achieving extremely high Q-factors of 120,000, indicating that innovative coupling techniques can successfully circumvent some traditional electrical limitations. Compounding these energy loss issues is the challenge of thermal stability. The inherent temperature coefficient of piezoelectric materials causes changes in elasticity and density, leading to thermal expansion that alters the SAW propagation velocity. This characteristic is a dual-edged sword: while it poses a significant stability problem for frequency-critical applications, it also enables the creation of highly sensitive temperature sensors, with measured sensitivities reaching as high as 116.685°/°C. Furthermore, a direct thermal impact on loss mechanisms is evident in piezo-active [110] GaAs cavities, where the Q-factor decreases linearly with temperature, a behavior directly correlated with the increased ohmic resistivity of the metallic reflectors. Addressing these fundamental limitations in energy dissipation and thermal drift, which are intrinsic to conventional materials and structures, therefore demands a radical shift toward novel materials and advanced fabrication paradigms capable of engineering acoustic properties at a more granular level.

Overcoming the intrinsic physical limitations of SAW technology requires moving beyond conventional fabrication paradigms and integrating novel materials, a shift that introduces its own significant engineering challenges in process control, material quality, and structural integrity. Additive manufacturing techniques, such as aerosol jet printing, present a compelling alternative to traditional cleanroom-based photolithography, offering a dramatic reduction in fabrication time from approximately 40 h to just 5 min. However, this advantage comes with critical trade-offs. The primary challenge is conductivity; the electrical conductivity of non-sintered, printed silver nanowires is lower by a factor of 45 compared to conventionally evaporated gold, a performance gap that necessitates post-processing steps like sintering. Furthermore, maintaining structural fidelity is difficult, as phenomena like “overspray” can compromise the precision of fine electrode features and negatively impact device performance.

The integration of novel 2D materials like MXenes introduces another layer of complexity. Here, the ability to precisely control surface termination chemistry is of paramount importance. Research has shown that oxygen-terminated Ti_3_C_2_T_x_ monolayers exhibit 20% higher SAW velocities than their fluorine-terminated counterparts, a difference attributed to stronger intralayer bonds. This extreme sensitivity makes termination uniformity a critical challenge for achieving predictable and repeatable device performance. The difficulties are amplified in multilayer MXene structures, where the transition from a single layer introduces new, low-frequency interlayer vibrational modes that cause increased wave dispersion and reduce the coherence of the propagating SAW. This rapid diversification in fabrication techniques and material systems, from printed conductors to 2D films, creates a divergence in device performance that makes direct comparison untenable, escalating the need for system-level integration strategies and a common framework of standardized metrics.

For SAW devices to be successfully deployed in real-world systems, they must not only perform well individually but also integrate seamlessly into larger RF architectures and be characterizable by a common set of metrics. This presents hurdles in both system design and industry-wide standardization. Critical RF design challenges for practical applications include achieving proper impedance matching for wireless power transfer, ensuring electromagnetic compatibility (EMC), and the co-design of antennas for integrated SAW-based tags. While these are crucial for system integration, the current body of research focuses primarily on device physics and materials science, indicating that these system-level engineering problems represent a vital and distinct area for future development. As the field explores a wider array of materials, fabrication methods, and device architectures, the need for standardized reporting of key performance indicators becomes increasingly urgent. Across the research landscape, a wide variation in performance is evident. The Q can range from approximately 6700 in some piezo-active cavities to a remarkable 120,000 in optically coupled systems. Similarly, designs may target a specific Fractional Bandwidth (FBW), such as the 2–5% required for certain 5G filters, while prioritizing low Insertion Loss (IL). A pivotal parameter, the Electromechanical Coupling Coefficient (K^2^), also varies dramatically, from 6.1% in lithium tantalate to a high of 13.8% for LLSAW modes on lithium niobate. This wide spectrum of reported values makes direct, objective comparison between different technologies exceedingly difficult and underscores the necessity for a standardized framework for evaluating and reporting device performance. By establishing this common language for performance, the field can more effectively architect a technology roadmap that leverages these disparate innovations toward unified, strategic goals.

Current research is actively paving the way for a technology roadmap focused on achieving all-optical control, enabling scalable manufacturing, and facilitating deep integration with emerging quantum systems. A key element of this vision is a paradigm shift toward optical-only SAW platforms, moving away from traditional electromechanical transducers toward coherent optical coupling methods, such as those leveraging Brillouin-like interactions. This approach is critical for accessing high-Q SAW modes on non-piezoelectric, quantum-relevant substrates like silicon and diamond, which are inaccessible with conventional techniques. This innovation is complemented by a trajectory toward the scalable, wafer-level printing of IDTs. Research into aerosol jet printing serves as a foundational step toward this goal, promising rapid, cleanroom-free fabrication that is essential for the mass production and commercialization of SAW technologies. Perhaps the most profound impact lies in the potential for creating hybrid quantum systems. With their low loss and strong coupling capabilities, SAWs are an exciting resource for interacting with a variety of quantum systems, including superconducting qubits, semiconductor quantum dots, color centers, and even superfluids. In this context, optically coupled SAWs provide an ideal pathway for realizing robust, long-distance quantum links. Ultimately, while formidable challenges in physics, fabrication, and integration persist, the convergent innovations in optical control, additive manufacturing, and quantum coupling are charting an unambiguous and transformative path. These efforts are positioning SAW technology to evolve from a mature RF component into a cornerstone of next-generation communication and quantum information systems.

## 7. Conclusions

SAW technology has evolved from a mature platform for analog signal processing and RF filtering into a rapidly diversifying field that now spans optics, electronics, magnetics, microfluidics, and even quantum science. This transformation is driven by the convergence of new physical mechanisms for SAW generation and control, reconfigurable device architectures, emerging material platforms, and agile fabrication methods that shorten the path from concept to demonstration. SAWs are no longer confined to strong piezoelectrics and fixed filters; instead, they increasingly act as tunable interfaces that mediate energy and information between different physical domains.

On the physics and device side, optical and magneto-acoustic techniques have expanded what SAWs can do and where they can exist. Brillouin-like optomechanical coupling and stimulated Brillouin scattering now enable SAW operation in non-piezoelectric and integrated photonic platforms, while magnetoelastic structures reveal nonreciprocal diffraction and spin–acoustic effects. In parallel, electrically reconfigurable SAW elements such as voltage-tunable phase shifters, compact UHF delay lines, and multi-stage lattice filters demonstrate how acoustic devices can be co-designed with electronics for adaptive RF and sensing functions. Materials such as thin-film piezoelectrics, engineered stacks on silicon and glass, and 2D systems like MXenes offer new levers to tailor velocity, attenuation, coupling, and dissipation, linking microscopic structure to macroscopic performance.

Advances in fabrication further reinforce this shift. Additive and maskless approaches, exemplified by aerosol jet-printed SAW microfluidic platforms, enable rapid prototyping and customization beyond the constraints of traditional cleanroom processing. SAW-assisted printing and particle patterning show that acoustic fields can also act as tools for assembling functional composites and soft robotic elements. Together, these developments are broadening the application space to include hybrid quantum interfaces, lab-on-a-chip manipulation, high-sensitivity sensing, reconfigurable communication front-ends, and acoustically driven actuators. Therefore, SAW technology is moving from a narrow RF role to a versatile, programmable platform, well positioned to serve as a bridging layer among electronic, photonic, mechanical, and quantum systems in next-generation hybrid technologies.

## Figures and Tables

**Table 1:** Comparative analysis of novel Surface Acoustic Wave (SAW) technologies.

Core Technology and Physical Mechanism	Device Configuration and Materials	Target Application	Key Performance Metrics	Novelty and Fabrication Highlights	Ref.
Aerosol Jet Printing (Additive Manufacturing) Utilizes acoustic radiation force and streaming for microfluidic manipulation.	Printed IDTs: Ag nanowires, Graphene, PEDOT:PSS inks. Substrate: 128° Y-cut LiNbO_3_.	Microfluidics (Streaming, particle concentration).	Freq: 6.95–19.92 MHz Streaming: ~550 μm/s Droplet: 5 μL	Rapid Prototyping: Reduces fabrication time from ~40 h (cleanroom) to ~5 min; maskless, direct-write process	[170]
Coherent Optical Coupling Brillouin-like optomechanical interaction mediated by Gaussian SAW cavity modes.	Fabry–Perot Cavity: Curved Al reflectors. Substrate: GaAs (including piezo-inactive cut).	Quantum Systems (Transduction, sensing, material spectroscopy).	Freq: ~500 MHz Q-Factor: ~120,000 (record high) Coupling ($g_{o}$): 2π × 1.4 kHz	Piezo-Independence: Enables high-Q acoustic modes in piezo-inactive directions; non-contact probing of dissipation.	[148]
Acoustoelectric Effect SAW electric fields couple with mobile carriers in a semiconductor to modulate velocity/loss.	Hybrid Structure: ALD-deposited ZnO Thin-Film Transistor (TFT) on 41° Y-cut LiNbO_3_.	Signal Processing (Reconfigurable Phase Shifters).	Freq: 455 MHz (LLSAW) Tuning: 1.22% velocity shift Atten: 8.02 dB/mm	Mode Optimization: Utilizes Longitudinal Leaky SAW (LLSAW) for higher coupling ($K_{eff}^{2}$ ≈ 13.8%) compared to Rayleigh modes.	[156]
Acoustic Wetting (PSLEA) Forms a micron-sized liquid film at the chip edge to separate atomization from droplet jetting.	Edge-Atomizer: Paper strip located at the edge of 128° Y-cut LiNbO_3_ chip.	Medical Devices (Nebulizers/Inhalation therapy).	Freq: ~30 MHz Rate: 2.6 mL/min Particle Size: 3.95 μm (median)	Thermal Management: Reduced maximum thermal stress by 45% (4.3 × 10^8^ N/m^2^) compared to surface loading.	[176]
Nonreciprocal Diffraction Resonant scattering via ferromagnetic resonance (magnon-phonon coupling).	Magneto-elastic Grating: Ni nanowires (110 nm thick) on Z-cut LiNbO_3_.	Microwave Comm. (Isolators, Circulators).	Freq: 2.6 GHz Asymmetry: ~2% intensity diff. (up vs. down).	Symmetry Breaking: First observation of diffraction intensity depending on magnetic field polarity; reversed by diffraction direction.	[141]
SAW-Assisted Printing Dual-field control: Acoustic radiation force (position) + Magnetic torque (orientation).	Composite Resin: Fe_3_O_4_ particles (1 μm) in elastic resin; patterned by SAW.	Soft Robotics (Magnetic gears, actuators).	Patterning: 200 μm spacing Response: 13.5% increase in magnetic responsiveness.	Multi-Field Control: Simultaneous spatial patterning and magnetic pole alignment improves mechanical performance.	[175]
Stimulated Brillouin Scattering (SAW-SBS) Overlap of optical evanescent field and surface acoustic wave.	Chalcogenide Waveguide: Ge_11.5_As_24_Se_64.5_ core (116 nm thick) on SiO_2_.	Sensing and Signal Processing (On-chip photonic circuits).	Freq: 3.81 GHz Gain: 203 W^−1^m^−1^ Linewidth: 20 MHz	New Regime: First experimental observation of on-chip SAW-SBS; enables SAW excitation in non-piezoelectric platforms.	[149]
UHF Reflective Delay Line Rayleigh wave reflection; sensitivity depends on delay time/phase change.	Z-Shaped Delay Line: 128° YX LiNbO_3_; 50 nm Au electrodes (350 nm pitch).	Temperature Sensing (Passive/Wireless).	Freq: 2.45 GHz Sensitivity: 116.685°/°C Linearity: 1.26% error	Miniaturization: Z-shaped path reduces chip size while maintaining high delay time and sensitivity.	[174]
Wireless Power Transfer (WPT) Inductive coupling matched to SAW resonance; acousto-thermal effects.	ZnO Thin Film (~4.5 μm) on glass substrate; Cu coils for WPT.	De-icing/Defogging and Monitoring.	Freq: ~9.88 MHz TCF: ~44 ppm/°C Eff: Optimized at 1 cm distance	Wireless Integration: Minimizes localized heating compared to wired connections; integrated passive monitoring and active de-icing	[178]
Lattice Vibrations (Simulation) Interlayer van der Waals bonding and intralayer stiffness.	2D MXene Films: Ti_3_C_2_T_x_ (T = O, F); 1 to 10 layers.	Material Design (Tunable SAW devices).	Velocity: O-term (~7.3 km/s) > F-term (~3.9 km/s) for monolayers.	Surface Chemistry: Revealed that surface termination and layer stacking (1 vs. 2 layers) drastically modulate SAW speed.	[165]
Lattice-Type Network Synthesis Optimization of BVD model parameters (pole-zero distribution).	Multi-Stage Lattice: 42° YX LiTaO_3_ ($K_{t}^{2}=6.1\%$).	5G RF Filters (Sub-6 GHz bands).	FBW: 2–5% Return Loss: >10 dB Rejection: >40 dB	Optimization: 3-stage lattice design methodology achieves high selectivity/rejection for specific 5G bands (e.g., n77, n78).	[178]

## Data Availability

No new data were created or analyzed in this study. Data sharing is not applicable to this article.
